# Transcatheter arterial chemoembolization plus sorafenib versus transcatheter arterial chemoembolization alone to treat advanced hepatocellular carcinoma: a meta-analysis

**DOI:** 10.1186/s12885-017-3707-5

**Published:** 2017-11-06

**Authors:** Rong Cai, Rongfeng Song, Pengfei Pang, Yan Yan, Yifeng Liao, Cuiling Zhou, Shuncong Wang, Xiuling Zhou, Huaping Wang, Hongyu Zhang, Huanhuan Sun, Haiqing Ma

**Affiliations:** 1 Department of Oncology, The Fifth Affiliated Hospital of Sun Yat-sen University, Zhuhai, Guangdong 519000 China; 2 Department of Gastroenterology, Cancer Hospital of Jiangxi Province, Nanchang, Jiangxi 330029 China; 3 Center for Interventional Medicine, The Fifth Affiliated Hospital of Sun Yat-sen University, Zhuhai, Guangdong 519000 China; 4 Guangdong Provincial Engineering Research Center for Molecular Imaging, Zhuhai, Guangdong 519000 China; 5 Institute of Interventional Radiology, Sun Yat-sen University, Zhuhai, Guangdong 519000 China

**Keywords:** Transcatheter arterial chemoembolization, TACE, Advanced hepatocellular carcinoma, Sorafenib, Clinical trial

## Abstract

**Background:**

Many studies have combined sorafenib with transcatheter arterial chemoembolization (TACE) to treat patients with advanced hepatocellular carcinoma (HCC), but the results are disputable. Thus, we conducted this meta-analysis to assess the efficacy and safety of the combination treatment in patients with advanced HCC.

**Methods:**

Clinical data were collected from a computer search of literature published from January 2009 to June 2016 in PubMed, Web of Science, the Cochrane Library, China National Knowledge Infrastructure (CNKI), Wan Fang and the China Science and Technology Journal Database (CSTJ). The final analysis included 14 studies and 1670 patients. The primary endpoints were overall survival (OS), the objective response rate (ORR) and the disease control rate (DCR).

**Results:**

The combination group exhibited significantly more improvement than the group treated with TACE alone in ORR (RR =1.62, 95% confidence interval (CI) = 1.34–1.94, *p* < 0.00001), DCR (RR = 1.43, 95% CI = 1.26–1.62, *p* < 0.00001), 0.5-year OS (OR = 2.60, 95% CI = 1.57–4.29, *p* = 0.0002) and 1-year OS (OR = 1.88, 95% CI =1.39–2.53, *p* < 0.0001). The incidence of adverse events from combination therapy was increased compared to that from treatment with TACE alone, and the most commonly reported adverse events were fatigue, hand-foot skin reaction and diarrhoea, which were bearable.

**Conclusions:**

The meta-analysis indicated that combination therapy is safe and efficient for clinical application.

## Background

Hepatocellular carcinoma (HCC) is the fifth most common malignancy worldwide with high disease incidence, and it is the third leading cause of cancer-related death [1, 2]. Recently, the number of HCC patients has increased every year, and effective measures to cure HCC are limited. Given its insidious onset, nonspecific symptoms and a difficulty to diagnose at early stages, most HCC patients are diagnosed with intermediate or advanced stage disease with distant metastasis. Therefore, few patients have the opportunity to undergo radical surgery [3, 4]. In patients with advanced HCC, safer and more effective therapies are urgently needed given its high occurrence and low survival rate.

As for advanced liver cancer patients who are not eligible for surgery, comprehensive treatment based on transcatheter arterial chemoembolization (TACE) remains a major intervention for advanced HCC patients. Although TACE treatment can control tumour development and prolong life, liver tissue deteriorates after repeated TACE treatment and chemotherapy, ultimately aggravating the condition. Simultaneously, because TACE can block the primary blood vessels of the liver tumour and cause a local anoxic environment, it promotes the activation of vascular endothelial growth factor (VEGF) and vascularization, subsequently leading to recurrence and metastasis [5, 6].

Sorafenib, an oral multi-targeted receptor kinase inhibitor, is the first drug proven to be effective for the systemic treatment of advanced HCC patients [7, 8]. Because of the complicated pathogenesis of liver cancer and the poor outcome of single-agent treatment, combination therapy is a promising strategy. Sorafenib plus TACE to treat HCC has gradually become concerning clinical issue. In recent years, numerous studies have explored the combination of sorafenib with TACE for patients with advanced HCC, but the results are disputable. Many studies have reported the promising application of combination treatment in HCC [9–11]. However, in a few studies, combination therapy did not lead to improved OS in advanced HCC patients [12, 13]. Therefore, to evaluate the efficacy and safety of sorafenib combined with TACE to treat advanced HCC, we conducted a meta-analysis assessing objective response rate (ORR), disease control rate (DCR), overall survival (OS) and adverse reactions.

## Methods

### Literature search

Clinical data from advanced HCC patients treated with TACE and sorafenib were collected from the electronic databases PubMed, Web of Science, the Cochrane Library, CNKI, Wan Fang and China Science and Technology Journal Database (CSTJ) from January 2009 to June 2016. In total, 680 studies were collected. After assessing the efficacy and safety of the combination therapy, 14 articles were ultimately included.

### Inclusion criteria

(1) Research subjects were diagnosed with advanced HCC by clinical and pathological assessment. Moreover, these patients were not eligible for surgical treatment. (2) Research subjects were recruited to a clinical case-control study and were assigned to the TACE plus sorafenib group or the TACE group randomly or based on their wishes. In the TACE group, patients received TACE combination chemotherapeutics, and the chemotherapeutic agents that were concurrently used were epirubicin, cisplatin, gemcitabine, doxorubicin, irinotecan and mitomycin. In the combination group, 400 mg of sorafenib was administered twice daily from 3 to 7 days after TACE until the disease progressed or the patient died. (3) Studies must be published, and the primary data from case-control or cohort studies must have been provided in the publication. (4) Studies providing original data concerning the ORR, DCR, survival rate and adverse reactions. The data were either reported in these studies or calculated.

### Exclusion criteria

(1) The original data were not suitable for analysis. (2) Meeting abstracts, case reports, editorials, reviews and other meta-analyses were not included. (3) Multiple publications, duplicate records and similar studies were excluded.

### Data extraction

Two researchers extracted the data and independently assessed the inclusion and exclusion criteria. If a disagreement occurred between the two researchers, a third researcher would assist. After screening and examining the selected studies, the following indices were collected: (1) Primary information, such as the first author’s last name, publication date, type of study and literature reference. (2) Baseline conditions, such as the age, numbers of patients and sex of the research objects. (3) Outcome indicators, such as treatment plans, intervention measures, effective rates, survival rates and adverse reactions.

### Quality assessment

The JADAD scale was used to evaluate the quality of the included studies: (1) The generation of a random sequence: Grade 2 indicates appropriate, grade 1 unclear and grade 0 impertinent; (2) Allocation concealment: grade 2 means appropriate, grade 1 unclear and grade 0 impertinent or unused; (3) Blind method: Grade 2 indicates appropriate, grade 1 unclear and grade 0 impertinent; (4) Withdrawal and exit: Grade 1 indicates described, and grade 0 indicates not described. Grade 1–3 refers to poor-quality studies, in which bias is more likely to occur; and grade 4–7 indicates high-quality studies, in which bias is less likely to occur [14].

### Statistical analysis

Review Manager Version 5.3 software, which was recommended by the Cochrane Collaboration, was used for meta-analysis. *P* < 0.05 indicates a significant difference. OR is the odds ratio, RR is the risk ratio, and OS was defined as the time from the beginning of treatment to the date of death or the date at which patients were last known to be alive. The ORR was the sum of the complete and partial response. DCR was the sum of the complete and partial response and stable disease. Moreover, the 95% confidence intervals (CIs) were calculated. First, these studies were tested for heterogeneity, and *I* ^*2*^ statistic and *P*-values were used in the assessment. If *P* ≤ 0.1 and *I* ^*2*^ ≥ 50%, there were significant differences, and the random effects model was utilised. If *I* ^*2*^ < 50% and *P* > 0.1, there were no significant differences, and the fixed effects model was applied. Publication bias was analysed using funnel plots.

## Results

### Search results

**Fig. 1:**
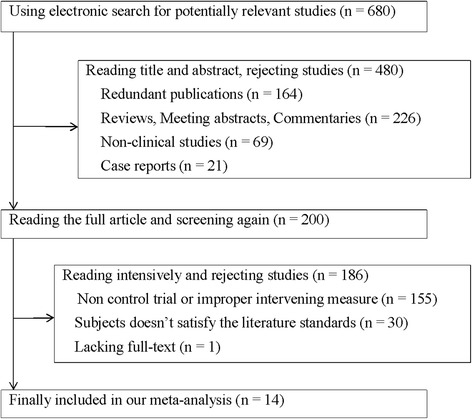
Flow diagram of the study selection process

According to the inclusion criteria, 14 studies and a total of 1670 patients with advanced HCC were included. The meta-analysis flow chart is shown in Fig. 1. No significant differences in age, sex and clinical stages were noted between the combination group (sorafenib combined with TACE) and the control group (TACE alone group) in the 14 included studies. The main characteristics are consistent between the two groups (Table 1).

**Table 1:** Main characteristics of the 14 studies included in the meta-analysis

		Patients (n)		Average age (year)		Treatment design		
Study	Type	Con (M/F)	Com (M/F)	Con	Com	Con	Com	JADAD score
Hu 2013 [28]	N	21/9	29/14	47.1	45.3	TACE	TACE+ Sorafenib	4
Wu 2010 [29]	N	21/4	20/5	–	–	TACE	TACE+ Sorafenib	6
Jiang 2010 [15]	R	23/7	24/6	58.0	56.0	TACE	TACE+ Sorafenib	4
Chen 2012 [30]	R	17/11	20/8	–	–	TACE	TACE+ Sorafenib	5
Wei 2009 [31]	N	22/8	24/6	–	–	TACE	TACE+ Sorafenib	5
Wei 2012 [16]	N	42/2	42/2	53.0	53.0	TACE	TACE+ Sorafenib	5
Yu 2011 [32]	R	20/5	21/4	45.3	45.3	TACE	TACE+ Sorafenib	5
Yang 2013 [33]	R	–	–	–	–	TACE	TACE+ Sorafenib	5
Ye 2013 [17]	N	30/5	32/3	–	–	TACE	TACE+ Sorafenib	5
Sun 2014 [34]	R	66/15	68/13	53.9 ± 8.2	54.5 ± 7.9	TACE	TACE+ Sorafenib	5
Zhou 2014 [35]	R	31/17	34/14	67.9 ± 10.8	71.9 ± 12.7	TACE	TACE+ Sorafenib	5
Wang 2015 [36]	R	28	22	–	–	TACE	TACE+ Sorafenib	5
Lencioni 2016 [13]	R	126/27	135/19	63.0	64.5	TACE	TACE+ Sorafenib	5
Kudo 2011 [26]	R	168/61	174/55	70.0	69.0	TACE	TACE+ Sorafenib	5

**Note**: A total of 1670 patients were included in the meta-analysis. Among these patients, 839 were assigned to the combination group (Com) and treated with TACE plus sorafenib, and 831 were assigned to the control group (Con) and treated with TACE alone

*Abbreviations*: *F* female, *M* male, *N* non-randomized controlled trials, *R* randomized controlled trials, and *-* no description

### Efficacy assessment

**Fig. 2:**
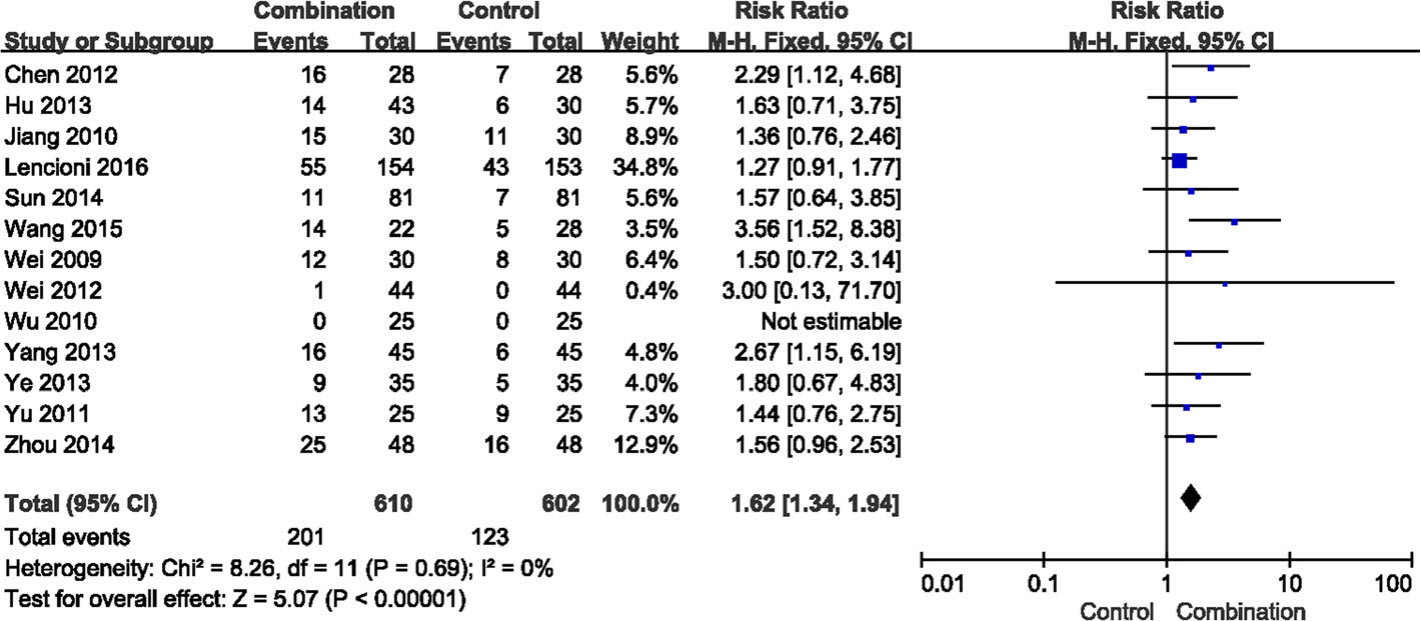
Forest plot comparing the objective response rate (ORR). CI, confidence interval; RR, risk ratio; Combination group, TACE + sorafenib; and Control group, TACE alone

**Fig. 3:**
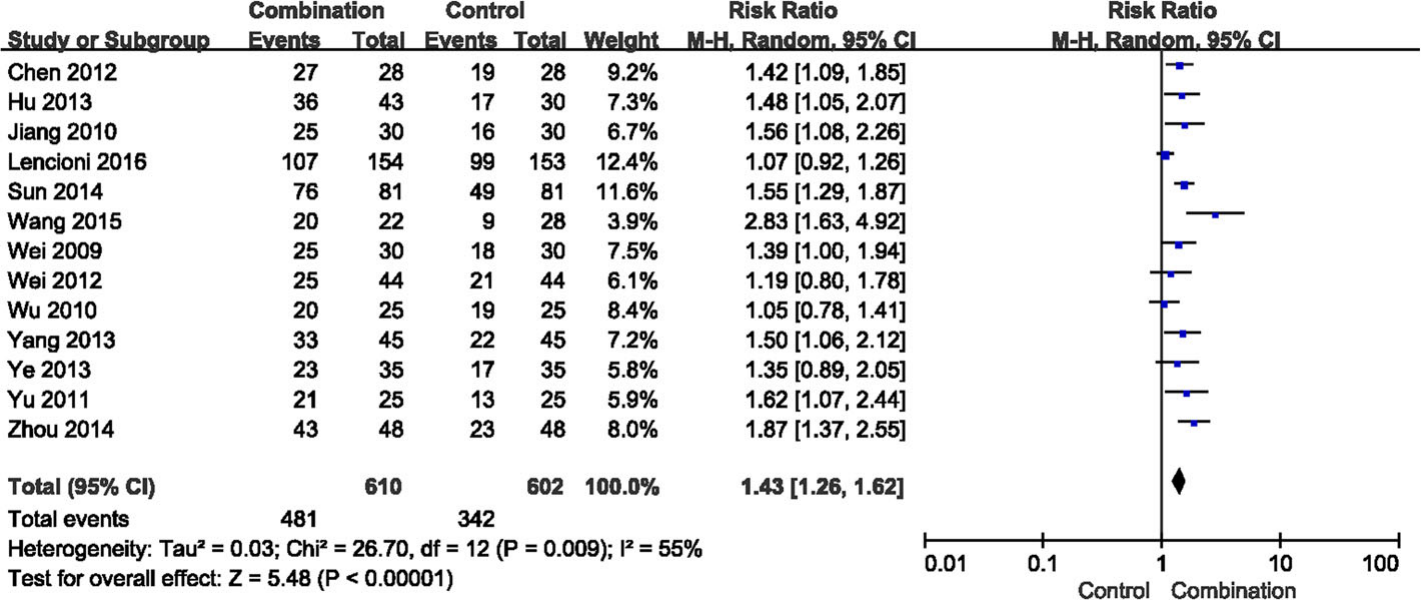
Forest plot comparing the disease control rate (DCR). CI, confidence interval; RR, risk ratio; Combination group, TACE + sorafenib; and Control group, TACE alone

Given the lack of heterogeneity, a fixed effects model was used to determine the RR for the ORR. The RR of the ORR among HCC patients was 1.62 (95% CI = 1.34–1.94, *p* < 0.00001) with no heterogeneity (*I* ^*2*^ *=* 0) (Fig. 2), indicating that the ORR was higher in the combination group than in the control group. In addition, the RR of DCR was 1.43 (95% CI = 1.26–1.62, *p* < 0.00001) with significant heterogeneity (*I* ^*2*^ *=* 55%) (Fig. 3) and suggested that the combination group might lead to improved DCR compared with the control group for advanced HCC. Here, the results showed that the combination of sorafenib and TACE was more effective than TACE treatment alone.

### Os

**Fig. 4:**
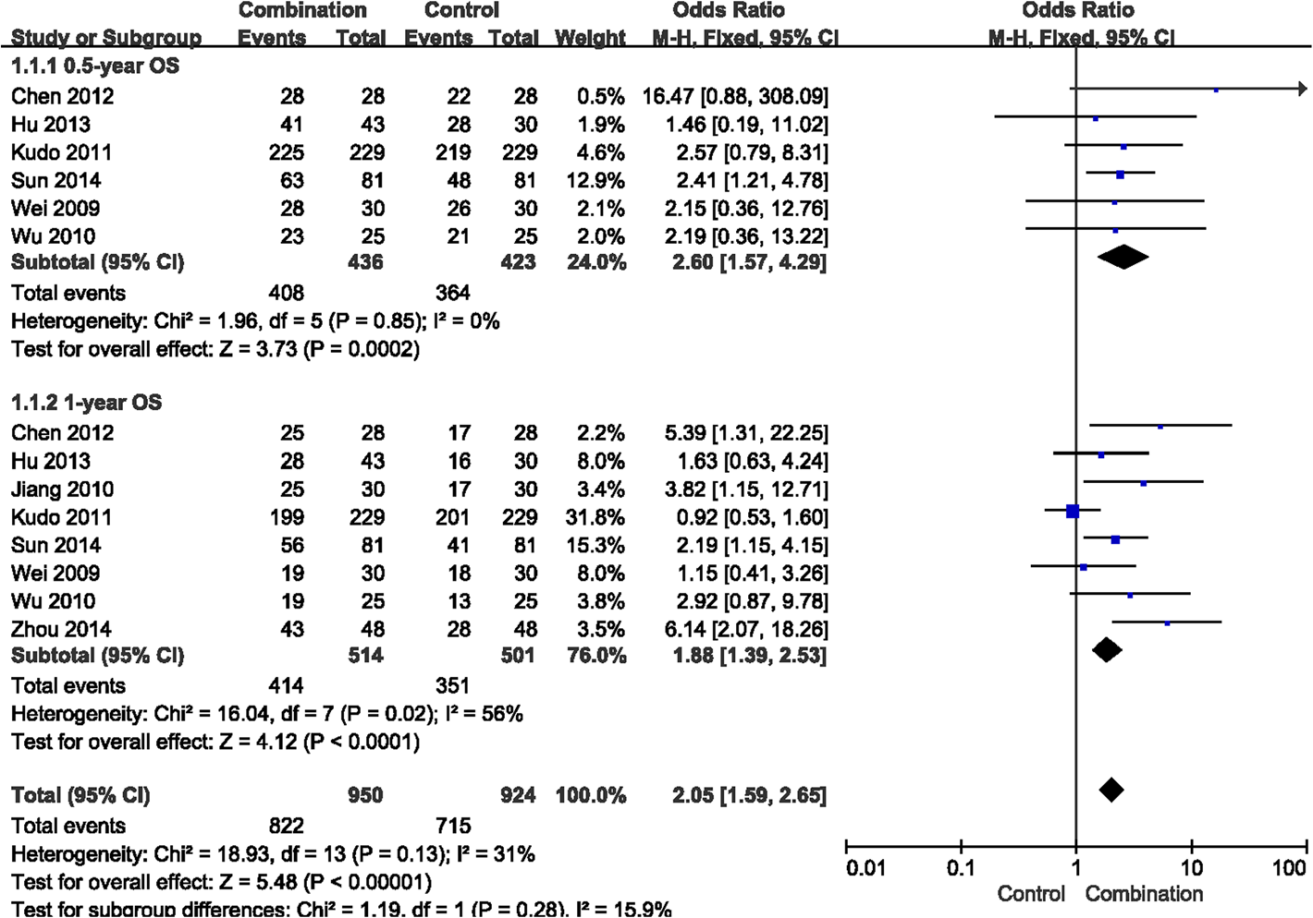
Forest plot comparing overall survival (OS). CI, confidence interval; OR, odds ratio; Combination group, TACE + sorafenib; and Control group, TACE alone

In the included studies, 8 presented available survival rate data. Low heterogeneity was noted between 0.5-year OS and 1-year OS after the heterogeneity test. Consequently, a fixed effects model was used in the meta-analysis. Combination therapy increased the 0.5-year OS (OR = 2.60, 95% CI = 1.57–4.29, *p* = 0.0002) (Fig. 4). Moreover, the 1-year OS in the combination group was (OR =1.88, 95% CI = 1.39–2.53, *p* < 0.0001) (Fig. 4). The results confirmed that the combination treatment significantly prolongs survival time.

### Median OS and median time to progression

In our study, 5 studies reported median OS and median time to progression (TTP). After the heterogeneity test, the result from Table 2 show that the median OS and median TTP were significantly increased in the combination group compared to the control group [15–17].

**Table 2:** Comparison of the median overall survival and median time to progression

Study	Median OS (month)		*P*	Median TTP (month)		*P*
	Com	Con		Com	Con	
Jiang 2010 [15]	–	–	–	6.2	3.1	< 0.001
Wei 2012 [16]	21.0	10.0	0.006	11.0	6.0	0.001
Ye 2013 [17]	14.8	8.2	0.023	10.3	5.8	0.035
Lencioni 2016 [13]	–	–	0.295	24.1 (wks)	23.7 (wks)	0.072
Kudo 2011 [26]	29.7	–	0.790	5.4	3.7	0.252

*Abbreviations*: *OS* overall survival, *TTP* time to progression, *Com* combination group, *Con* control group, and *-* no description

### Adverse reactions

**Fig. 5:**
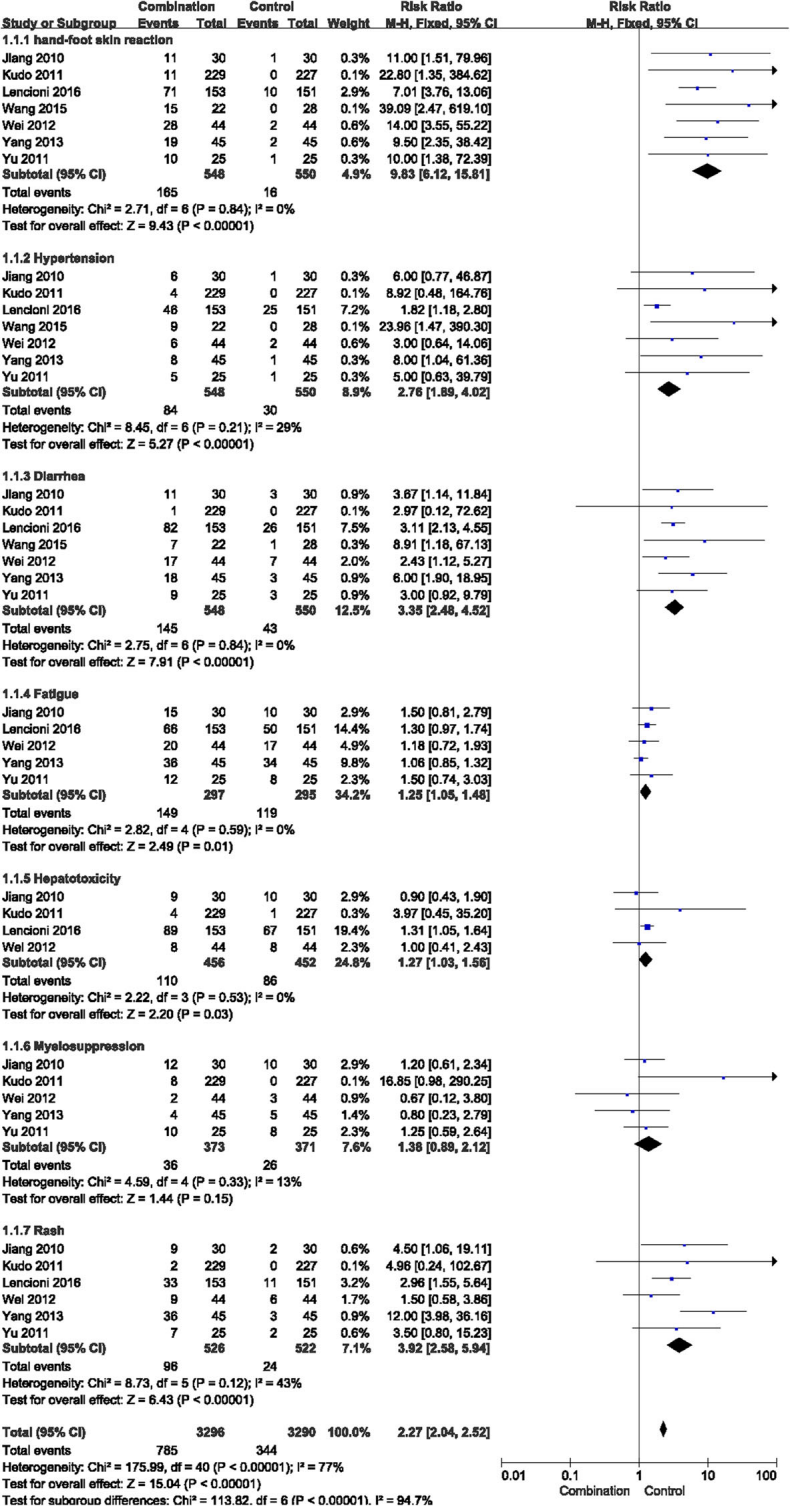
Forest plot comparing toxicity (hand-foot skin reaction, hypertension, diarrhoea, fatigue, hepatotoxicity, myelosuppression and rash). CI, confidence interval; RR, risk ratio; Combination group, TACE + sorafenib; and Control group, TACE alone

**Fig. 6:**
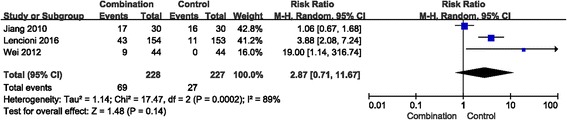
Forest plot comparing toxicity (alopecia). CI, confidence interval; RR, risk ratio; Combination group, TACE + sorafenib; and Control group, TACE alone

Seven studies reported that two methods led to adverse reactions in patients with advanced HCC, especially in the combination group. The primary adverse reactions were fatigue, hand-foot skin reaction, diarrhoea, hypertension, hepatotoxicity, alopecia, myelosuppression and rash. The fixed effects model was used to analyse hand-foot skin reaction, hypertension, diarrhoea, fatigue, hepatotoxicity, myelosuppression and rash (Fig. 5), whereas the random effects model was used to analyse alopecia (Fig. 6).

The incidence of hand-foot skin, hypertension, diarrhoea, fatigue, hepatotoxicity and rash were significantly increased for combination treatment compared with that for TACE alone (RR = 9.83, 95% CI = 6.12–15.81, *p* < 0.00001; RR = 2.76, 95% CI =1.89–4.02, *p* < 0.00001; RR = 3.35, 95% CI =2.48–4.52, *p* < 0.00001; RR = 1.25, 95% CI = 1.05–1.48, *p* = 0.01; RR = 1.27, 95% CI = 1.03–1.56, *p* = 0.03; and RR = 3.92, 95% CI = 2.58–5.94, *p* < 0.00001, respectively).

The incidence of myelosuppression and alopecia did not significantly increase in the combination treatment group compared with the TACE alone group (RR = 1.38, 95% CI =0.89–2.12, *p* = 0.15; and RR = 2.87, 95% CI =0.71–11.67, *p* = 0.14, respectively).

To a certain extent, the combination therapy increased the incidence of adverse reactions. However, there were no serious reactions in the referenced studies, and these reactions could be alleviated to different degrees after symptomatic treatment. Hence, combination therapy was a relatively safe option to treat advanced HCC.

### Publication bias

**Fig. 7:**
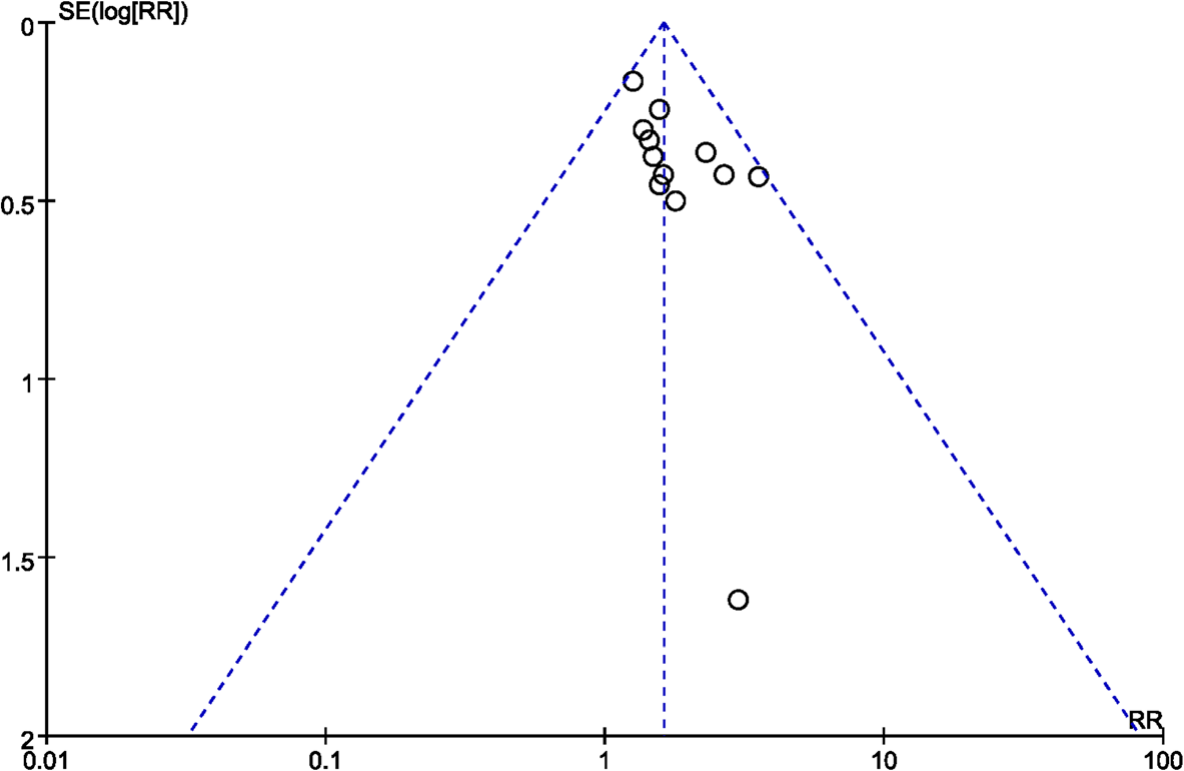
Funnel plot comparing the objective response rate (ORR). CI, confidence interval; RR, risk ratio; Combination group, TACE + sorafenib; and Control group, TACE alone

The funnel plot was applied to resolve the publication bias for this meta-analysis. Figure 7 indicates that the comparison of ORR was among the 95% confidence intervals. In addition, the scatter points were distributed symmetrically in the inverted funnel. All the evidence indicates that the probability of publication bias is low.

## Discussion

This meta-analysis provides comprehensive data to evaluate the treatment effects and safety of sorafenib combined with TACE to treat advanced HCC patients. The 14 studies used in the meta-analysis included randomized controlled trials and non-randomized controlled trials, which provided relatively safe and accurate evidence for the final clinical data. Compared with the single TACE treatment group, the data indicate that advanced patient prognosis is better after sorafenib and TACE combination therapy, as illustrated by increased ORR, DCR and OS. Although the probability of adverse reactions was increased with combination therapy, the side effects could be relieved by reducing the dose or suspending treatment.

The 14 included studies provided raw experimental ORR and DCR data for the meta-analysis. The research data indicate that combination therapy significantly increases the ORR and DCR of advanced HCC patients. All results mentioned above were statistically significant. The 0.5-year OS values from 6 studies were compared, and 8 studies were used to compare one-year OS. The meta-analysis results indicate that the 0.5-year OS of the combined treatment was 93.58%, whereas it was 86.05% in the TACE alone group. Moreover, the one-year OS was 80.54% in the combined treatment group, whereas for the TACE alone group it was 70.06%. Sorafenib combined with TACE clearly increased the 0.5-year and 1-year OS for advanced HCC patients. This study shows that compared with TACE alone, combination therapy has a more positive survival rate outcome. Low heterogeneity was noted between the two groups, indicating the comparability of data and the reliability of the conclusion.

Early diagnosis was halted in the majority of patients due to the insidious and nonspecific clinical manifestation of HCC. As a result, most cases are diagnosed at advanced HCC stages and received only topical treatment or palliative care services, including TACE, surgical operation, radiofrequency ablation and anhydrous alcohol injection therapy [18]. However, these local treatments promote VEGF activation, an important angiogenic factor that promotes division and migration and also supports endothelial cells [19]. Thus, increasing the VEGF concentration might cause adverse outcomes. Previous research has shown that VEGF expression in HCC is increased 7-fold compared with that in normal hepatocytes [20]. Therefore, it is extremely important to seek new therapeutic methods to increase clinical effectiveness and prolong survival for HCC patients.

Sorafenib, a multi-target oral medicine and multi-enzyme inhibitor, inhibits liver cancer, advanced renal carcinoma, melanoma, and non-small-cell lung cancer. Sorafenib inhibits HCC growth by inhibiting tumour cell proliferation and angiogenesis [21, 22]. Previous studies revealed that TACE and sorafenib might act synergistically to inhibit HCC growth via different mechanisms. Because of poor prognosis and the lack of effective treatments for HCC, combination therapy is urgently required. In recent years, many studies have shown benefits from the combined use of TACE plus sorafenib for advanced HCC [23, 24], but a few studies have raised questions. For example, a meta-analysis published in Hepatology International in 2016 showed that the combination therapy did not improve OS (HR = 0.79, *p* = 0.235) in advanced HCC patients [12]. Similarly, a SPACE trial published in the Journal of Hepatology in 2016 also showed that the combination treatment did not improve OS (HR 0.898, *p* = 0.29) [13]. Our study confirmed that the combination treatment is a practicable measure for HCC patients by evaluating ORR, DCR and OS. During TACE treatment, sufficient treatment intervals must be provided to allow liver function recovery before the next treatment, because chemotherapy drugs affect liver function [25]. As shown in Table 2, 3 studies reported that TACE treatment combined with sorafenib significantly prolonged the median TTP [15–17], but the other 2 studies did not indicate long-term tumour stability with combination treatment [13, 26].

Of the included studies, seven studies reported that both therapies caused adverse reactions during advanced HCC treatment, including fatigue, alopecia, hand-foot skin reaction, diarrhoea, hypertension, hepatotoxicity, myelosuppression, and rash [27]. The two groups exhibited significant differences in the incidence of hand-foot skin reaction, rash, diarrhoea, fatigue, hepatotoxicity and hypertension but not myelosuppression and alopecia. Analysing the data for adverse reactions, the Chi-square of alopecia was 17.47, via homogeneity analysis, indicating substantial heterogeneity for this adverse reaction, which could be caused by the smaller number of research studies. Combination therapy increased the occurrence rate of adverse reactions compared with that for the control group. However, most of these adverse events were relieved after reducing the dose or symptomatic treatment. Generally, combination therapy is a relatively safe treatment.

Sorafenib is an important supplement to TACE, which fails to remove the tumour. To date, the treatment effects and safety of combination TACE with sorafenib treatment has been observed in advanced HCC patients. Several studies have presented similar results as ours; however, our study collected more comprehensive tests to improve statistical reliability. Our results are consistent with the results of the included studies. Thus, TACE plus sorafenib is an effective treatment for advanced HCC patients.

However, there are several limitations to our study. First, the follow-up time was not sufficient. Second, the data we analysed were extracted from published papers rather than original patient records, which could lead to bias in the analysis results and influence the accuracy of our conclusion. Third, this study demonstrated that the 0.5-year OS and 1-year OS could be improved by combination treatment, but a longer survival time was not indicated. Therefore, additional studies are required to confirm the safety and efficacy of combination therapy.

## Conclusions

In summary, this study shows that combination therapy significantly increased ORR, DCR and OS, verifying its efficiency. In addition, adverse reactions can be alleviated. Therefore, combination therapy of sorafenib plus TACE for advanced HCC is an optimal and safe treatment for patients.

## Abbreviations

CI: Confidence interval
DCR: Disease control rate
HCC: Hepatocellular carcinoma
OR: Odds ratio
ORR: Objective response rate
OS: Overall survival
RR: Risk ratio
TACE: Transcatheter arterial chemoembolization
TTP: Time to progression
